# Induced Neurons From Germ Cells in *Caenorhabditis elegans*

**DOI:** 10.3389/fnins.2021.771687

**Published:** 2021-12-03

**Authors:** Iris Marchal, Baris Tursun

**Affiliations:** ^1^Berlin Institute for Medical Systems Biology, Berlin, Germany; ^2^Max Delbrück Center for Molecular Medicine in the Helmholtz Association, Berlin, Germany; ^3^Department of Biology, Institute of Zoology, University of Hamburg, Hamburg, Germany

**Keywords:** germline, neuron, reprogramming, epigenetics, chromatin, safeguarding, *C. elegans*

## Abstract

Cell fate conversion by the forced overexpression of transcription factors (TFs) is a process known as reprogramming. It leads to de-differentiation or *trans-*differentiation of mature cells, which could then be used for regenerative medicine applications to replenish patients suffering from, e.g., neurodegenerative diseases, with healthy neurons. However, TF-induced reprogramming is often restricted due to cell fate safeguarding mechanisms, which require a better understanding to increase reprogramming efficiency and achieve higher fidelity. The germline of the nematode *Caenorhabditis elegans* has been a powerful model to investigate the impediments of generating neurons from germ cells by reprogramming. A number of conserved factors have been identified that act as a barrier for TF-induced direct reprogramming of germ cells to neurons. In this review, we will first summarize our current knowledge regarding cell fate safeguarding mechanisms in the germline. Then, we will focus on the molecular mechanisms underlying neuronal induction from germ cells upon TF-mediated reprogramming. We will shortly discuss the specific characteristics that might make germ cells especially fit to change cellular fate and become neurons. For future perspectives, we will look at the potential of *C. elegans* research in advancing our knowledge of the mechanisms that regulate cellular identity, and what implications this has for therapeutic approaches such as regenerative medicine.

## Introduction

### Transcription Factor-Induced Reprogramming of Cell Fates

Forced overexpression of transcription factors (TFs) can induce reprogramming to dedifferentiate or *trans-*differentiate mature cells. Thereby, either induced pluripotent stem cells, or other specific types by direct conversion can be generated, respectively (Yamanaka, 2012; Wang et al., 2021). The prospect that reprogrammed cells could be used for tissue replacement therapies to repair diseased or injured tissues in patients demands for efficient reprogramming procedures. Yet, TF-induced reprogramming is often restricted and depends on the context of tissue types (Brumbaugh et al., 2019; Haridhasapavalan et al., 2020). As a consequence, TF expression that can induce ectopic fates in highly plastic cells, such as in early embryos, usually fail to reprogram mature cells in a complex adult multicellular organism (Yuzyuk et al., 2009; Tursun et al., 2012). The limitation of TFs to convert cell fates is caused by factors that safeguard cellular identity and prevent perturbations of their state. Understanding the molecular mechanisms that are involved in cellular fate safeguarding provides insight into what defines cell types at the molecular level and illustrates which factors are crucial in the correct transition from one type to the other (Rothman and Jarriault, 2019). The germline of *C. elegans* helped identifying a number of evolutionarily conserved factors that act as barriers for TF-induced reprogramming of germ cells to neurons, which will be summarized in this review.

### The *Caenorhabditis elegans* Germline: Specification, Proliferation and Differentiation

**FIGURE 1:**
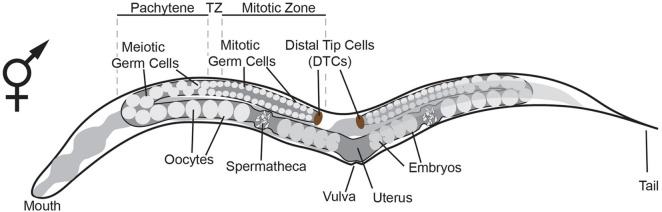
Schematic representation of the adult hermaphrodite germline. The germline consists of two gonadal arms, with each arm containing mitotic germ cells, meiotic germ cells, oocytes and spermatheca. The somatic distal tip cells (DTCs) are located at the distal most end of the gonad where they control germline mitosis (Byrd et al., 2014). As germ cells move away from the DTC and reach the transition zone (TZ), they enter and proceed through meiosis. After the TZ, cells move through the pachytene where they grow until they enter the proximal arm as oocytes. Oocyte maturation is triggered by the spermatheca (Miller et al., 2001). The oocyte enters the spermatheca at ovulation and is fertilized giving rise to an embryo.

During *C. elegans* development, the germline is set apart from the soma by the 16–24 cell stage of embryogenesis (Sulston et al., 1983). At that stage, germline potential is appointed to the P blastomeres which ultimately give rise the first primordial germ cell (PGC) P4. By the time the nematode has reached the adult stage, PGC P4 has proliferated and given rise to an adult germline of over a thousand cells in the hermaphrodite (Hirsh et al., 1976). An adult *C. elegans* hermaphrodite germline consists of two gonadal arms, with each arm containing mitotic stem cells, meiotic cells, oocytes and sperm cells (Figure 1). The somatic distal tip cells (DTCs) are located at the distal most end of the adult gonad where they control germline mitosis and thereby provide the niche for adult germ line stem cells (GSC) (Byrd et al., 2014). As germ cells move away from the DTC and reach the transition zone, they enter and proceed through the different stages of meiotic prophase I (Hirsh et al., 1976). After the transition zone, cells move through the pachytene where they gradually grow until they enter the proximal arm as oocytes. As *C. elegans* is a hermaphrodite, oocyte maturation is triggered by sperm-derived major sperm protein (MSP) and happens to the oocyte closest to the spermatheca (Miller et al., 2001). Subsequently, the oocyte enters the spermatheca at ovulation and is then fertilized, giving rise to a whole new organism (McCarter et al., 1999).

## Safeguarding Germline Identity by Repressing Unsolicited Induction of Neuronal Fates

With its property of giving rise to meiotic cells, the *C. elegans* gonad provides a unique possibility to study molecular mechanisms that maintain totipotency and that protect the germ cell fate. The totipotency and immortality of the germline is protected by preventing differentiation toward somatic fates. This safeguarding is controlled at multiple levels from translational modifications to post-transcriptional regulation and through extensive chromatin regulation.

### Safeguarding Germline Identity by Regulating Protein Translation

At the protein translation level, two conserved translational regulators, MEX-3 and GLD-1, are essential for maintaining totipotency. Ciosk et al. (2006) showed that in a *mex-3 gld-1* double mutant germ cells spontaneously differentiated into somatic cell types, including two types of muscle (pharynx and body), unspecific neurons and intestinal cells. The induction of the mixed somatic fates is accompanied by tissue type-specific characteristics. These include filaments and adhesive structures resembling those found in normal muscles, pan-neuronal *unc-119:GFP* reporter expression typical for neurons and auto-fluorescent granules similar to those of wild-type intestinal cells (Ciosk et al., 2006). The somatic differentiation as observed in the *mex-3 gld-1* double mutants is reminiscent of human germ cell tumors called teratomas consisting of mixed tissue types (Ciosk et al., 2006). Moreover, *mex-3 gld-1* double mutants show a significant reduction in size and number of germ cell-specific P-granules in the central regions of the germline. P-granules are specialized ribonucleoprotein structures and their reduction is likely to be a hallmark of germ cells that undergo differentiation toward somatic fates.

### Safeguarding Germline Identity by P Granules

Interestingly, it was later shown that P granules provide another level of germline protection, as loss of P granules by itself may cause differentiation of germ cell into somatic lineages (Updike et al., 2014). Germline specific P-granules, also known as germline granules, are composed of two main classes of RNA-binding proteins belonging to the RGG domain-containing proteins: PGL-1 and PGL-2, and the DEAD-box proteins GLH1-4 (Jennifer and Wang, 2014; Strome and Updike, 2015). The role of P-granules in cell fate regulation was revealed when simultaneous depletion of PGL-1 and PGL3 in combination with GLH-1 and GLH-4 induced expression of the body wall muscle myosin (MYO-3) and the pan-neuronal reporters *unc-119:GFP* and *unc-33:GFP* (Updike et al., 2014). The GFP-expressing germ cells had extended neurite-like projections suggesting differentiation into specific neuronal subtypes. However, expression of neuronal markers that report terminally differentiated neurons was not observed. Interestingly, promoting the terminal differentiation of neurons toward the glutamatergic taste neuron identity through additional ectopic expression of the fate-inducing Zn-finger TF CHE-1 (Tursun et al., 2012) did result in the expression of the terminal ASE neuron fate marker *gcy-5:GFP* (Updike et al., 2014). Overall, these results show that P-granules act as a barrier for differentiation of germ cell to somatic cell types through their role in small RNA biogenesis and post-transcriptional regulation, thereby maintaining the totipotency of germ cells.

### Preventing Unsolicited Induction of Neuronal Fates at the Epigenetic Level

**FIGURE 2:**
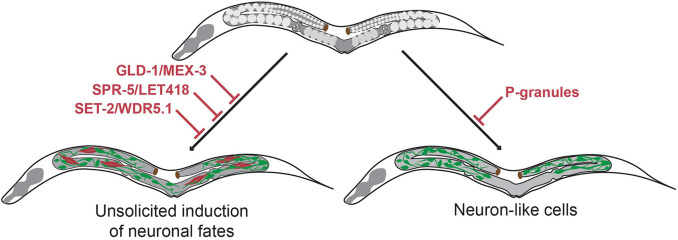
Molecular mechanisms that maintain germline totipotency and prevent unsolicited induction of somatic fates. Upon loss of the translational regulators GLD-1 and MEX-3 germ cells spontaneously differentiate to somatic cells thereby forming germline teratomas that contain multiple cell types at once including neurons (Ciosk et al., 2006). Loss of the chromatin regulators SPR-5/LET-418 (Käser-Pébernard et al., 2014) and SET-2/WDR-5.1 (Robert et al., 2014) also result in germline teratomas. Loss of germline P-granules leads to differentiation of germ cells to neuron-like cells, which do no acquire characteristics of terminally differentiated neurons (Updike et al., 2014). Green cells indicate neuron-like cells, red cells indicate muscle-like cells.

Another level of protection of germline totipotency is located at the level of epigenetics. Suppression of the evolutionary conserved chromatin regulators SPR-5 and LET-418 (the worm homologs of Lysine-specific histone demethylase (LSD-1) and Mi2 respectively) causes *C. elegans* germ cells to display teratoma-like characteristics (Käser-Pébernard et al., 2014). Germ cells express pan-neuronal genes such as *unc-119*, obtain neuron-like projections or express muscle markers such as MYO-3 (myosin) in *spr-5 let-418* double mutants (Käser-Pébernard et al., 2014). The demethylase SPR-5 interacts with LET-418 in two complexes, the nucleosome remodeling and deacetylase (NuRD) complex and the MEC complex. Hence, the absence of SPR-5 allows increased H3K4 methylation, indicating increased chromatin activation (Käser-Pébernard et al., 2014). Moreover, another study showed that knock-out of the H3K4 methyltransferase SET domain-containing 2 (SET-2) or its cofactor, WD-repeat 5.1 (WDR-5.1), also leads to expression of somatic markers in the germline and causes soma-like differentiation of germ cells (Robert et al., 2014). Again, this somatic differentiation was characterized by expression of neuronal genes such as *ceh-2* and *ceh-20* and muscle genes such as *unc-120*. These findings illustrate that loss of epigenetic regulators and altered chromatin regulation affect the epigenetic landscape of the germline and provide a permissive context for spontaneous germ cell transdifferentiation into somatic cell lineages (Figure 2).

## Overcoming Barriers of Transcription-Factor Mediated Germ-Cell-To-Neuron Reprogramming

### The Histone Chaperone LIN-53 Prevents Transcription-Factor Mediated Germ-Cell-to-Neuron Reprogramming

Cellular transdifferentiation by the forced overexpression of cell-fate inducing TFs is limited due to cell fate safeguard mechanisms. As described above, these protective mechanisms often rely on epigenetic regulation. As a result, TFs that can induce ectopic fates in highly plastic cells such as developing embryos, usually fail to induce conversion of germ cells to somatic identities (Tursun et al., 2012). One factor that has been identified as a barrier for neuronal induction in germ cells is the histone chaperone LIN-53 (Harrison et al., 2006). LIN-53 prevents direct reprogramming of germ cells into ASE neurons upon heat-shock induced overexpression of the zinc finger TF CHE-1 (Tursun et al., 2012). While overexpression of CHE-1 alone in embryos resulted in the ectopic expression of the ASE fate marker *gcy-5:GFP* in most embryonic cells, broad CHE-1 mis-expression in adult worms allowed marker expression only in a small number of head sensory neurons but nowhere else in the animal.

**FIGURE 3:**
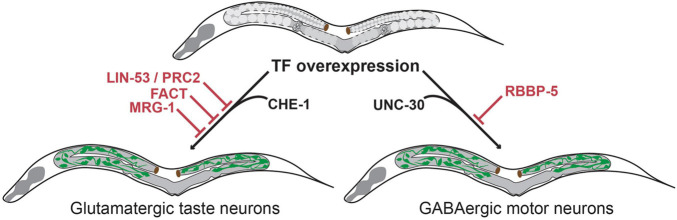
Transcription factor-induced germ cell reprogramming to neuronal fates. The histone chaperone LIN-53 prevents reprogramming of germ cells into glutamatergic taste neurons (known as ASE) upon overexpression of the Zn-finger TF CHE-1 (Tursun et al., 2012). LIN-53 cooperates with the Poly Comb Repressive Complex 2 (PRC2) to prevent neuronal induction in the germline (Patel et al., 2012). The heterodimeric histone chaperone FACT (Kolundzic et al., 2018) and the chromodomain protein MRG-1 block germ cell conversion to glutamatergic neurons (Hajduskova et al., 2019). The methyltransferase complex member RBBP-5 blocks germ cell reprogramming to GABAergic neurons upon UNC-30 TF-induction (Kazmierczak et al., 2020).

RNAi mediated knock-down of *lin-53* in combination with CHE-1 overexpression in adult animals allowed induction of *gcy-5:GFP* in mitotic germ cells. The converted germ cells expressed markers for the pan-neuronal fate (e.g., *rab-3*, *unc-119*, *snb-1*, *unc-33* and *unc-10*) as well as for the specific neuron sub-type (*gcy-5*, *ceh-36* and *eat-4*), while expression of markers for other neuron sub-types were not observed. Moreover, the converted cells underwent drastic morphological changes adopting neuron-like nuclear morphology and growing axonal projections (Tursun et al., 2012). These morphological changes were accompanied by loss of P-granules and of PGL-1, illustrating a complete conversion of germ cells into neuron-like cells. *lin-53* removal also permitted conversion of germ cells into other neuron sub-types. Upon overexpression of the EBF-like TF UNC-3 or the Pitx type homeodomain TF UNC-30 germ cells were converted to cholinergic or GABAergic motor neurons respectively. Like CHE-1 induction, UNC-3 or UNC-30 induction resulted in germ cells losing their characteristic morphology, adopting neuron-like morphology and growing axonal projections. Interestingly, converted germ cells displayed neuronal identity markers that are corresponding to the specific fate that is induced by the overexpressed TF. For example, in the case of UNC-3 induced reprogramming converted cells only express a marker for cholinergic ventral cord motor neurons (*acr-2*) but none of the ASE neuronal fate markers. These observations indicate that this conversion is different from undirected differentiation of germ cells into mixed somatic cell types as observed during teratoma formation. Instead, the TF-induced conversion upon depletion of LIN-53 is specific and directed toward distinct neuron sub-types depending on the overexpressed TF. Notably, although the mis-expression of the cell fate inducing TFs (CHE-1, UNC-3 and UNC-30) and the RNAi mediated depletion of *lin-53* were both in the entire adult body, neuronal induction occurs in the germline only. Hence, removal of LIN-53 allows direct conversion into distinct neuronal subtypes in what seems to be a germline-specific manner (Figure 3).

Interestingly, the CAF-1 histone chaperone complex (containing the mouse ortholog of LIN-53) was later identified as a strong cellular safeguard of somatic cell identity during reprogramming to neurons and induced pluripotent stem cells (iPSCs) (Cheloufi and Hochedlinger, 2017), indicating that the role of LIN-53 as a reprogramming barrier is conserved.

### LIN-53 Cooperates With PRC2 to Prevent Neuronal Induction in the Germline

LIN-53 is a component of many distinct multiprotein complexes (e.g., NuRD, CAF, HAT1 and PRC2 complex) (Loyola and Almouzni, 2004; Harrison et al., 2006) with various functions in chromatin biology. Further study showed that the effect of *lin-53* depletion in germ-cell-to-neuron reprogramming is phenocopied by the removal of other components of the Polycomb repressive complex 2 (PRC2) (Patel et al., 2012; Figure 3). PRC2 is a highly conserved epigenetic regulator that represses chromatin through the deposition of H3K27 di- and trimethylation marks, which are associated with developmentally regulated genes (Kelly and Fire, 1998; Xu et al., 2001). This observation suggests that PRC2 defines a chromatin state that protect the genome from aberrant regulatory inputs. Disruption of this chromatin state in germ cells renders them susceptible to direct reprogramming into neurons. Interestingly, this protective chromatin state may differ among cell types as the loss of PRC2 only allows induction of neuronal and muscle fate in germ cells, but no other somatic cell types (Patel et al., 2012).

### The Methyltransferase Complex Member RBBP-5 Blocks Transcription Factor-Induced Conversion of Germ Cells to GABAergic Neurons

The methyltransferase complex member RBBP-5 was recently identified as a novel germ cell reprogramming barrier in a screen to identify factors that increase LIN-53 depletion mediated reprogramming efficiency into GABAergic neurons (Kazmierczak et al., 2020). Although LIN-53 depletion alone allows germ cell to GABAergic neuron reprogramming upon UNC-30 overexpression, the conversion efficiency is rather limited when compared to conversion to glutamatergic ASE neurons upon CHE-1 overexpression. To test whether GABAergic neuron induction could be enhanced, additional chromatin regulators were tested in co-depletion with LIN-53. This led to the identification of RBBP-5 as a novel reprogramming barrier that blocks conversion of germ cells specifically into GABAergic neurons (Figure 3).

The mechanisms by which RBBP-5 operates as a barrier to reprogramming remains to be determined. However, these results illustrate the high specificity of the molecular programs that define cellular sates and antagonize the induction of neuronal cell fates.

### The FACT Complex Member HMG-3 Is a Conserved Reprogramming Barrier in the Germline

To reveal other factors that are barriers to neuronal reprogramming in *C. elegans*, Kolundzic et al. (2018) performed a whole-genome RNAi screen using overexpression of the fate inducing TF CHE-1 and identified around 119 target genes that allow ectopic *gcy-5:GFP* induction in the germline upon depletion (out of 171 total targets identified as reprogramming barriers, other tissues tested were intestine, muscle and epidermis). These factors are implicated in a number of biological processes such as proteostasis, cell shape, mitochondrial function, aging and nuclear factors (Kolundzic et al., 2018).

Among the candidates identified as barriers to neuronal reprogramming were the three subunits of the histone chaperone FACT (Facilitates Chromatin Transcription) namely HMG-3, HMG-4 and SPT-16 (Kolundzic et al., 2018). The study showed that FACT has two tissue-specific isoforms in *C. elegans*. HMG-3 is exclusively expressed in the germline, where it forms a complex with the ubiquitously expressed SPT-16. In contrast, HMG-4 is predominantly expressed in the soma and thereby forms the somatic isoform of FACT together with SPT-16.

As a result, RNAi-mediated depletion of *hmg-4* and *spt-16* allowed partial intestine-to neuron reprogramming, whereas depletion of *hmg-3* allows germ-cell-to-neuron conversion. Interestingly, single-molecule fluorescence *in situ* hybridization (smFISH) revealed that intestinal cells switch to a stable neuron-like gene expression profile upon *hmg-4* and *spt-16* depletion- mediated reprogramming. However, the converted cells do not obtain a neuron-like morphology. Yet, depletion of *hmg-3* allows extended conversion into neurons as illustrated by changes in nuclear morphology and the expression of multiple pan-neuronal and neuron-specific reporter genes (pan-neuronal: *rab-3*, *unc-119*; ciliated neurons: *ift-20*, ASE-expressed neuronal genes (*gcy-5*, *ceh-36*, *rab-3*, *unc-10*, and *unc-119*). Notably, depletion of germline-specific FACT without CHE-1 induction led to an impairment of cell fate maintenance of the germline. Depletion of *hmg-3* decreased the expression of germ-cell specific markers such as PIE-1, and P-Granule levels indicating that the permissiveness for germ cell to neuron reprogramming upon *hmg-3* RNAi is created by weakening the starting cell fate.

The same study also demonstrated that FACT’s function as a reprogramming barrier is conserved, as siRNA mediated depletion of the human FACT homologs SSRP1 and SUPTH16 enhanced reprogramming efficiency of human fibroblasts into iPSCs and induced neurons (Kolundzic et al., 2018). Chromatin and transcriptome analysis upon FACT knockdown revealed a general decrease in expression of factors previously described as reprogramming inhibitors such as CAF-1 (Cheloufi and Hochedlinger, 2017) and an increase in reprogramming promoting factors such as SALL4 (Buganim et al., 2013). Taken together, FACT is an evolutionary conserved reprogramming barrier that safeguards cellular identity by maintaining appropriate gene expression profiles (Figure 3).

### The Chromodomain Protein MRG-1 Blocks Transcription Factor Mediated Neuronal Induction in Germ Cells

MRG-1 is a component of the NuA4 histone acetyl transferase complex and is orthologous to the mammalian chromodomain-containing MRG15 (Chen et al., 2009). It has been shown to regulate the proliferation and differentiation of *C. elegans* germ cells during development (Fujita et al., 2002; Gupta et al., 2015). MRG-1 was recently identified as another novel factor that counteracts germ cell reprogramming into neurons upon neuron-fate inducing TF overexpression (Hajduskova et al., 2019). RNAi mediated depletion of *mrg-1* in combination with CHE-1 expression allowed germ-cell-to neuron conversion. As described before for LIN-53 and FACT, the converted neurons obtained molecular and morphological characteristics resembling the specific neuronal fate. Neuronal expression was confirmed by both transgenic reporter expression and smFISH. Moreover, the reprogrammed germ cells lost their P-granules and PIE-1 expression indicating a faithful conversion of germ cells into neurons.

The function of MRG-1 as a reprogramming barrier in the germline is independent from that of LIN-53 and PRC2. Whereas depletion of *lin-53* and other members of the PRC2 complex leads to global loss of H3K27me3, there were no changes in this chromatin mark observed in *mrg-1*-depleted animals. In fact, ChIP-seq analysis of MRG-1 showed very limited colocalization with LIN-53 and instead showed that it primarily binds loci that carry the active chromatin marks H3K36me3, H3K9ac and H3K4me3 (Hajduskova et al., 2019). This finding suggests that MRG-1 might protect the germline from converting into neurons by maintaining the expression of germline-specific genes (Figure 3).

Interestingly, immunoprecipitation of MRG-1 followed by mass-spectrometry (IP-MS) identified SIN-3, SET-26 and OGT-1 as novel interacting partners. This finding indicates that MRG-1 might also be involved in repressive chromatin regulating complexes. Since these interaction partners all mediate chromatin regulation, they might contribute to MRG-1’s function as cellular safeguard of the germline. Indeed, *sin-3*, *set-26* and *ogt-1* mutants increase reprogramming efficiency upon *mrg-1* depletion, indicating that these factors cooperate with MRG-1 in preventing neuronal induction in the germline (Hajduskova et al., 2019). Overall, MRG-1 seems to act as a safeguard of active chromatin signature to maintain germ cell identity—at the same time it cooperates with repressive chromatin regulators to prevent ectopic gene expression.

A more recent study performing in-depth CoIP-MS additionally detected a strong interaction of MRG-1 with the Small Ubiquitin-like Modifier (SUMO) (Baytek et al., 2021). It was shown that MRG-1 is post-translationally modified by SUMO, and that this modification affects the chromatin binding profile of MRG-1. SUMO has been implicated in stabilizing cell identity in the context of reprogramming somatic cells to iPSCs (Cossec et al., 2018). Moreover, SUMOylation of the TF Gatad2a, a component of the nucleosome remodeling and deacetylase (NuRD) complex, disrupts the assembly and stability of NuRD thereby inhibiting iPSCs formation (Mor et al., 2018). However, it is not yet known whether this extra layer of regulation through the SUMOylation of MRG-1 has an effect on its function as a barrier of germ cell to neuron reprogramming.

### Mammalian Germ Cell to Neuron Reprogramming

Recent studies in mammals have investigated the use of germline stem cells (GSCs) as a potential source of neuronal tissues for clinical therapy (Chen et al., 2020). For example, it was shown that functional neurons and glia cells can be generated from adult mouse spermatogonial stem cells (SSCs) (Glaser et al., 2008; Streckfuss-Bömeke et al., 2009). Established protocols for neural differentiation of murine embryonic stem cells (ESCs) using fibroblast growth factor (FGF) and sonic hedgehoc (shh) expression as initiation for differentiation were adapted to differentiate GSCs into neurons and glia cells (Glaser et al., 2008; Streckfuss-Bömeke et al., 2009). The GSC-derived neuron populations contain specific subtypes (including GABAergic, glutamatergic and dopaminergic) and show membrane potential properties and postsynaptic currents resembling fully functional matured neurons *in vitro* (Glaser et al., 2008; Streckfuss-Bömeke et al., 2009).

More recently, Yang et al. (2019) generated functional dopaminergic (DA) neurons from human spermatogonial stem cells (hSSCs). To convert hSSCs to neurons they were exposed to olfactory ensheathing cell conditioned culture medium (OECCM) containing FGF and shh and additional small molecules such as forskolin, valproic acid and SB431542 (Yang et al., 2019). The exposure to these small molecules has previously been used to differentiate mouse embryonic fibroblasts (MEFs) to neural crest like precursors (Takayama et al., 2017) and was shown to be of critical importance to achieve hSSCs to DA neuron conversion (Yang et al., 2019). SSC-derived DA neurons obtained gene-expression profiles similar to wild type DA neurons and acquired neuronal morphological features. Moreover, they obtained sophisticated functional properties typical for DA neurons including synapse formation, dopamine release, spontaneous action potentials and neuron-specific calcium flux. When the SSC-derived DA neurons were implanted in the striatum of a mouse model of Parkinson disease (PD) they survived, migrated, and further converted into DA neurons without causing tumor formation. Strikingly, transplantation of the SSC-derived DA neurons into the PD mouse model improved sensorimotor function (Yang et al., 2019). This observation supports the therapeutic potential of germline-derived neurons in treating neurogenerative diseases.

Generally, clinical application of GSCs is beneficial when compared to embryonic stem cells as it bypasses ethical concerns, risk of teratoma formation and immune rejection (Yamanaka, 2020). Studies in mammalian systems illustrates the relevance of fundamental research into cellular reprogramming in *C. elegans*, as mechanisms are conserved and analogous across species and findings can provide new avenues for future regenerative medicine applications.

## Future Perspectives

In this review, we have discussed our current knowledge regarding cell fate safeguard mechanisms and the molecular mechanisms underlying TF-mediated reprogramming of *C. elegans* germ cells into neurons. Detailed molecular and morphological analyses have shown an ability to reprogramming germ cells into multiple specific neuronal subtypes upon depletion of reprogramming barriers. The studies described here, mainly focus on factors involved in chromatin regulation. At the level of chromatin regulation, numerous factors have been identified as reprogramming barriers that seem to act in separate pathways. This indicates the multiple independent levels of protection of cells to safeguard their identities. Moreover, the whole-genome RNAi screen by which FACT was identified as a cellular safeguard revealed other candidates implicated in multiple biological processes such as proteostasis, cell shape, mitochondrial function, and aging (Kolundzic et al., 2018). It will be fascinating to reveal the molecular mechanisms by which these factors regulate cell fate and whether their role in blocking reprogramming into neurons is evolutionary conserved. Also, better understanding the molecular mechanisms of cell fate protection and reprogramming blocking may explain why some barriers appear to be tissue or context-specific. It is important to determine which barrier factors are expressed in which cell types—and importantly—at which transcript and protein levels. The levels and availability of required partnering factors may also vary in different tissues. It is conceivable that varying expression levels in different cell types and availability of required complex members (for LIN-53 e.g., subunits of PRC2, NuRD, CAF, or SIN3) (Loyola and Almouzni, 2004; Harrison et al., 2006) may influence the strength of a factor as a reprogramming barrier. Future studies in combination with single-cell transcriptome and genome analyses will provide more insight to which degree safeguarding factors and barriers are restricted to protecting certain tissue types at the functional level or simply due to limited availability.

As shown by the depletion of the FACT complex members *hmg-4* and *spt-16*, stable changes in gene expression profiles toward the new fate is not always sufficient to obtain fully induced neurons to an extent where they possess neuron-like morphology. Interestingly, germ cell reprogramming does not seem to suffer from this issue. Additionally, most reprogramming barriers are expressed in multiple tissues and their depletion in combination with fate-inducing TF expression was performed in the whole organism. However, primarily germ cells appear to be the tissue that allows full neuronal induction. This raises the question whether reprogramming mechanisms differ between cell types, and whether germ cells possess any cell type-specific characteristics that make them particularly suited to change cellular fate.

One aspect that influences reprogramming with regard to final identity, specifically toward neurons, might be the very specific morphology changes needed. For example, some tissues, like the intestine, might be unfit for full conversion because of structural constraints. Moreover, we could speculate that the initial function of the germline could influence the ability to reprogram. The unique feature of totipotency in the germline might provide protection strategies that are distinct from somatic tissues, which need to maintain a specific differentiated state. Alternatively, the intrinsic cellular context, mode of metabolism, and the micro- and macro-environment of the starting cell type might make specific cell types particularly amenable for reprogramming (Rothman and Jarriault, 2019; Lambert et al., 2021).

So far, studying the extend of reprogramming of induced neurons from germ cells has mainly focused on molecular and morphological features. Future analyses could be extended with functional assays such as electrophysiology to study whether they are capable of action potentials and network formation. Moreover, recent technological advancements at the single cell level (such as transcriptome and chromatin accessibility analyses) will allow us to study direct reprogramming more dynamically. Applying single cell technologies such as scRNA-seq and scATAC-seq for *C. elegans* will advance our knowledge of germline totipotency and mechanisms of germ cell safeguarding. Understanding these mechanisms will also improve techniques for generating neuronal tissues for clinical applications and might shed light on why some germ cells are well suited to become neurons while other cell types are not.

## Author Contributions

IM conceptualized and wrote the manuscript together with BT. BT helped to conceptualize the manuscript, advised and supported the manuscript writing. Both authors contributed to the article and approved the submitted version.

## Conflict of Interest

The authors declare that the research was conducted in the absence of any commercial or financial relationships that could be construed as a potential conflict of interest.

## Publisher’s Note

All claims expressed in this article are solely those of the authors and do not necessarily represent those of their affiliated organizations, or those of the publisher, the editors and the reviewers. Any product that may be evaluated in this article, or claim that may be made by its manufacturer, is not guaranteed or endorsed by the publisher.
